# Anxa4 mediated airway progenitor cell migration promotes distal epithelial cell fate specification

**DOI:** 10.1038/s41598-018-32494-z

**Published:** 2018-09-25

**Authors:** Kewu Jiang, Zan Tang, Juan Li, Fengchao Wang, Nan Tang

**Affiliations:** 1 College of Life Sciences, Beijing Normal University, Beijing, 100875 China; 2 National Institute of Biological Sciences, Beijing, 102206 China

## Abstract

Genetic studies have shown that FGF10/FGFR2 signaling is required for airway branching morphogenesis and FGF10 functions as a chemoattractant factor for distal epithelial cells during lung development. However, the detail downstream cellular and molecular mechanisms have not been fully characterized. Using live imaging of *ex vivo* cultured lungs, we found that tip airway epithelial progenitor cells migrate faster than cleft cells during airway bud formation and this migration process is controlled by FGFR2-mediated ERK1/2 signaling. Additionally, we found that airway progenitor cells that migrate faster tend to become distal airway progenitor cells. We identified that *Anxa4* is a downstream target of ERK1/2 signaling. *Anxa4*^−/−^ airway epithelial cells exhibit a “lag-behind” behavior and tend to stay at the stalk airways. Moreover, we found that *Anxa4*-overexpressing cells tend to migrate to the bud tips. Finally, we demonstrated that Anxa4 functions redundantly with Anxa1 and Anxa6 in regulating endoderm budding process. Our study demonstrates that ERK1/2/Anxa4 signaling plays a role in promoting the migration of airway epithelial progenitor cells to distal airway tips and ensuring their distal cell fate.

## Introduction

The airways of mammalian lungs are generated via a process called branching morphogenesis. Fibroblast growth factor 10 (FGF10) and its receptor FGFR2b are known to be essential for airway branching morphogenesis^1,2^; both *Fgf10*-null and *Fgfr2b*-null mouse embryos have a lung agenesis phenotype^3–5^. *Fgf10* is expressed in the distal mesenchyme, whereas *Fgfr2b* is uniformly expressed in the airway epithelium. As airway branching morphogenesis proceeds, *Fgf10* is dynamically expressed in the distal mesenchyme prior to the appearance of each new airway bud^6^. FGF10 has been shown to induce lung endoderm bud expansion and budding in mesenchyme-free lung endoderm explant cultures^6,7^. It has also been shown *in vitro* that FGF10 acts as a chemoattractant factor for distal airway epithelium^8,9^. These findings have established an essential role of FGF10 in regulating the directional outgrowth of airway buds during branching morphogenesis. However, the underlying cellular and molecular mechanisms through which FGF10 regulates airway bud formation are not well understood.

It is now appreciated that airway branching morphogenesis requires epithelial-mesenchymal interactions. In response to growth factors that are expressed in the mesenchyme (*e.g*., FGF10), epithelial cells initiate several processes including cell proliferation and cell migration^10,11^. A previous study showed that inhibition of cell proliferation in cultured chicken lung explants did not block new bud formation^12^ and this observation is consistent with another study which reported that localized proliferation is not a triggering event for the initiation of bud outgrowth in cultured lung endoderm explants^13^. However, multiple lines of evidence have demonstrated that cell migration is required for branching morphogenesis in several organs. Specifically, in the *Drosophila* trachea system, Bnl/Btl (homologs of FGF/FGFR) signaling controls trachea cell migration and branching morphogenesis^14^. It has been demonstrated that MAPK-dependent collective cell migration drives the branching morphogenesis and tube elongation of mammary gland^15,16^. During renal branching morphogenesis, GDNF-Ret signaling is known to be essential for the competitive cell migration: *Ret*^−/−^ cells exhibit a “lag-behind” behavior and contribute to trunks of ureteric bud^17,18^.

During airway branching morphogenesis, the airway epithelial progenitor cells in the distal tips differentiated into distinct cell lineages along its proximal-distal axis. The expression of Sox2 marks the proximal/stalk airway epithelial cell lineage whereas the expression of Sox9 and Id2 mark the distal airway epithelial cell lineage. Recent lineage-tracing studies using *Id2*^CreER/+^ mouse line show that airway epithelial progenitor cells in the distal bud tips are able to generate both distal and stalk airway epithelial cells at least up to E13.5^19^. It has been showed that FGF10 signaling is essential for preventing distal airway progenitor cell from differentiating into stalk airway epithelial cell^20,21^. However, it is less clear how airway branching morphogenesis is orchestrated with the airway epithelial cell fate specification.

Annexin proteins are found in species ranging from fungi to higher vertebrates. They are a highly-conserved superfamily of proteins that bind with membrane phospholipids in a calcium-dependent manner and this binding links them to many membrane-related processes (*e.g*., basic membrane organization, membrane trafficking)^22–24^. Further, it has been shown that several members of the Annexin family are able to bind with and “bundle” F-actin filaments^25–28^. *In vitro* studies including both gain- and loss-of-function experiments have shown that Annexins play a role in promoting cell migration^29,30^. Despite the abundance and conservation of Annexins in most eukaryotic species, relatively little is known about the regulation of *Annexin* gene expression and little is known about the function of Annexin proteins during embryonic lung development.

Here, using a combination of live imaging, mouse genetics and lung endoderm culture system experiments, we found that tip airway epithelial progenitor cells migrate faster than cleft cells during airway bud formation. We identified *Anxa4* (encoding Annexin A4) as a downstream target of ERK1/2 signaling and found that the expression level of *Anxa4* is positively regulated by the activity of ERK1/2 signaling. We showed that Anxa4 is required for airway epithelial cell migration, both *in vitro* and *in vivo*. Furthermore, we found that *Anxa4*^−/−^ epithelial cells exhibit a “lag-behind” behavior and tend to contribute to the stalk airways. In contrast, *Anxa4*-overexpressing cells tend to remain in bud tips during bud formation. We also found that Anxa4 functions redundantly with Anxa1 and Anxa6 in regulating the endoderm budding process. Our study establishes that FGF10-activated ERK1/2/Anxa4 signaling plays a role in promoting airway progenitor cell migration and ensuring their distal airway epithelial cell fate by regulating *Anxa4* expression during airway bud formation.

## Results

### Airway progenitor cells that migrate faster tend to commit to distal airway cell fate

**Figure 1:**
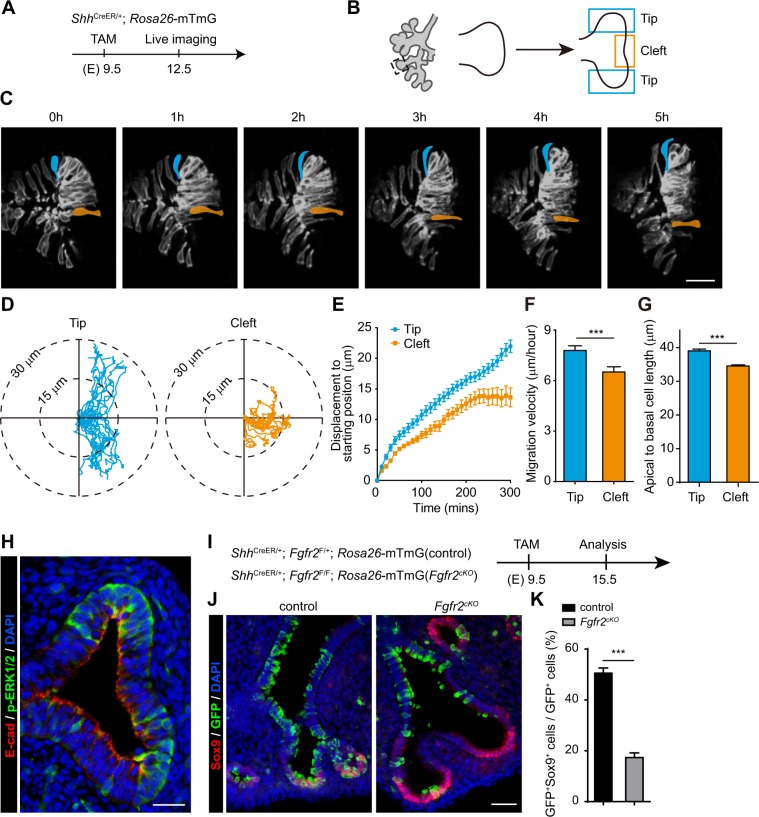
Airway progenitor cells that migrate faster tend to commit to distal airway cell fate. (**A**–**C**) Pregnant females carrying *Shh*^CreER/+^; *Rosa26*-mTmG embryos were treated with tamoxifen (TAM) at E9.5 and lungs were imaged at E12.5 (**A**). Bud tip of the first lateral branch in right caudal lobe (RCd.L1) was imaged. Finally, two new bud tips were formed while the middle cells between two bud tips formed the cleft (**B**). After five-hour imaging, the tip cells (one cell was highlighted by blue) directly moved out and formed a new bud tip, while the middle cells (one cell was highlighted by orange) moved slower and tended to lag behind (**C**). Scale bar: 50 μm. (**D**–**G**) Cell track plots of tip cells (24 cells) and cleft cells (17 cells) after aligning their starting positions (**D**), showing that tip cells can migrate longer than cleft cells (**D**,**E**); The migration velocity analysis showed that tip cells migrate faster than cleft cells (**F**); and the tip cells have longer apical-to-basal cell length (**G**). Data are presented as mean ± SEM; n = 3 live image samples; ***p < 0.001; Student’s *t*-test. (**H**) WT lung bud at E12.5 was analyzed by E-cadherin (red) and p-ERK1/2 (green) immunostaining. The bud tips showed higher p-ERK1/2 staining compared to cleft cells. Scale bar: 25 μm. (**I**–**K**) Pregnant females carrying *Shh*^CreER/+^; *Fgfr2*^*F/*+^; *Rosa26*-mTmG (control) and *Shh*^CreER/+^; *Fgfr2*^*F/F*^; *Rosa26*-mTmG (*Fgfr2*^*cKO*^) embryos were treated with tamoxifen (TAM) at E9.5 and lungs were sampled at E15.5 (**I**). Immunostaining using antibodies against GFP and Sox9 showed that less GFP^+^Sox9^+^ cells were detected in *Fgfr2*^*cKO*^ lung as compared to control lung (**J**,**K**). Data are presented as mean ± SEM; ***p < 0.001; Student’s *t*-test. Scale bar: 25 μm.

To investigate the cellular behaviors during airway bud formation, we conducted an *ex vivo* time-lapse imaging experiment with E12.5 lungs to monitor cell behaviors during airway bud formation. Pregnant females carrying *Shh*^CreER/+^; *Rosa26-*mTmG embryos were injected with one dose of tamoxifen at E9.5 (Fig. 1A). After a tamoxifen injection, membrane GFP was expressed in a mosaic manner in the airway epithelial cells of *Shh*^CreER/+^; *Rosa26-*mTmG lung, enabling tracing of individual airway epithelial cells. Airway epithelial progenitor cells at the tip of the first lateral branch in the right caudal lobe (RCd.L1) of lung at E12.5 were imaged (Fig. 1B). In wild type mice, this lateral branch undergoes a planar bifurcation that leads to the formation of two new buds^31^. The region between these two newly formed bud tips is referred to as the cleft (Fig. 1B). The RCd.L1 bud tip was imaged every 10 minutes for 5 hours. After five-hour live imaging, airway progenitor cells at the bud tip directly migrated out and formed a new bud tip, while airway progenitor cells at the cleft region migrated slower and formed the cleft (Fig. 1C and Supplementary Video 1).

To analyze cell migration of airway progenitor cells, we manually traced the positions of GFP cells at 31 time-points over a 5-hour-time-course live imaging by using Imaris software. Monitoring of the position data for each cell at each time points allowed us to directly analyze the migration tracks of cells and quantify both cell migration displacement and cell migration velocity (Fig. s1A). The cell migration displacement was calculated as the linear distance (in μm) between the current position of a given cell at a given time point and its starting position. The cell migration velocity was calculated as the ratio of cell migration distance (in μm) to migration time (in hours) (Fig. s1A,B). By analyzing migration tracks of airway progenitor cells at bud tip (tip cells) and airway progenitor cells at cleft region (cleft cells), we found that the tip cells migrated over longer distance and more directionally as compared to cleft cells (Fig. 1D). The average migration displacement over the 5 hours of the experiment was higher for tip cells as compared to cleft cells (Fig. 1E). Moreover, we found that the tip cells have higher migration velocity and longer apical-to-basal cell length as compared to cleft cells (Fig. 1F,G).

We next sought to identify the molecular mechanisms underlying our observation that tip cells migrate faster than cleft cells during planar bifurcation. Given that ERK1/2 signaling can be activated by FGF10/FGFR2 signaling and is essential for cell migration during development^32,33^, we hypothesized that FGF10/FGFR2 may regulate cell migration of tip cells via ERK1/2 signaling during airway bud formation. Indeed, the level of p-ERK1/2 was higher in cells of the two nascent bud tips than in cells of the cleft region during the planar bifurcation process, suggesting that ERK1/2 signaling may control tip cell migration (Fig. 1H).

Similar to lung, kidneys are highly branched organs and renal branching morphogenesis is known to requires GDNF and its receptor Ret^34,35^. It has been demonstrated that Ret is required for directed movement of progenitor cells during renal development and *Ret*^−/−^ cells tend to lag behind and contribute to the renal trunks^17^. To investigate the effect of loss of FGFR2 signaling on cell migration, we conditionally knocked out *Fgfr2* in airway epithelial cells at E9.5 using *Shh*^CreER/+^ mice and live imaged the RCd.L1 bud tips of *Shh*^*CreER/*+^; *Fgfr2*^*F/F*^; *Rosa26-*mTmG (*Fgfr2*^*cKO*^) lungs at E12.5 (Fig. s1C and Supplementary Video 2). We found that *Fgfr2*^−/−^ cells showed shorter migration track and smaller migration displacement than control cells and that their migration velocity was much slower than control cells (Fig. s1D–F and Supplementary Video 2). Additionally, we found that some *Fgfr2*^−/−^;GFP^+^ cells underwent apoptosis (Fig. s1G), consistent with previous study that loss of *Fgfr2* induces cell death^36^.

We next investigated the effect of loss of *Fgfr2* on airway epithelial cell fate determination. Based on the patterns of gene expression, stalk and distal airway epithelial cells can be distinguished by the expression of Sox2 or Sox9. We quantified the ratio of GFP^+^Sox9^+^ cells to total GFP^+^ cells in either *Shh*^*CreER/*+^; *Fgfr2*^*F/*+^; *Rosa26-*mTmG (control) or *Shh*^*CreER/*+^; *Fgfr2*^*F/F*^; *Rosa26-*mTmG (*Fgfr2*^*cKO*^) lungs at E15.5 (Fig. 1I). We found that fewer GFP^+^ cells were present in distal airways of *Fgfr2*^*cKO*^ lungs than in control lungs (Fig. 1J,K). Collectively, our findings indicate that Fgfr2 controls distal airway cell fate commitment by regulating ERK1/2-signaling-controlled cell migration.

### ERK1/2 signaling regulates the expression of *Anxa4*

**Figure 2:**
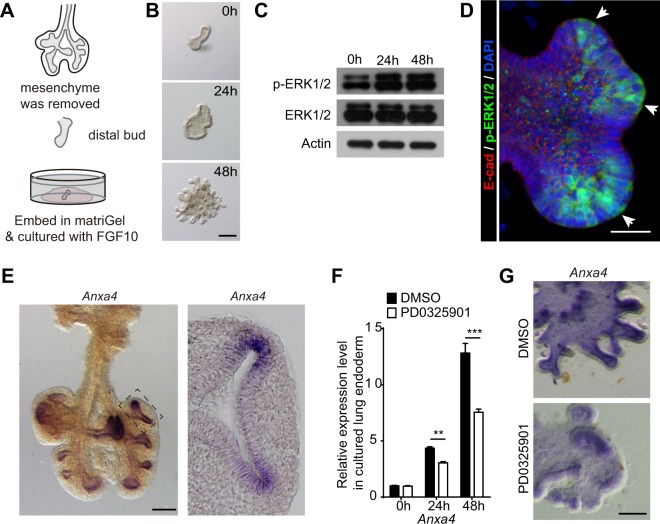
ERK1/2 signaling regulates the expression of *Anxa4*. (**A**) Diagram illustrating the method for mesenchyme-free lung endoderm culture. Whole lung at E11.5 was digested with trypsin on ice and then the mesenchyme was removed using fine forceps to get intact lung endoderm. The distal lung endoderm buds were dissected out, embedded in Matrigel and cultured in serum-free medium containing recombinant FGF10. (**B**) Images of cultured lung endoderm taken at different time points. There is no obvious bud formation at 24 h after culture. By 48 h, the lung endoderm starts budding and forms many buds. Scale bar: 200 μm. (**C**) Western blot of cultured lung endoderm explants at 0 h, 24 h and 48 h using antibodies against p-ERK1/2, total ERK1/2 and reference Actin. The phosphorylation level of ERK1/2 was increased significantly at both 24 h and 48 h. Full-length blots are presented in Supplementary Fig. s6. (**D**) Bud of cultured lung endoderm were analyzed by E-cadherin (red) and p-ERK1/2 (green) immunostaining. The bud tips (arrowhead) showed higher p-ERK1/2 staining compared to middle cells. Scale bar: 25 μm. (**E**) Whole-mount and section *in situ* hybridization of *Anxa4* in WT lungs at E12.5. *Anxa4* was highly expressed in the bud tip epithelial cells adjacent to FGF10 expressing site. Scale bar: 200 μm. (**F**,**G**) The expression level of *Anxa4* in cultured lung endoderm explants at 0 h, 24 h and 48 h. The expression level of *Anxa4* was increased significantly at both 24 h and 48 h as compared to 0 h and its expression level was inhibited by MEK inhibitor, PD0325901 (**F**). The whole mount *in situ* hybridization of *Anxa4* confirmed that PD0325901 treatment could decrease the expression of *Anxa4* (**G**). Data are presented as mean ± SEM, n = 3; **p < 0.01; ***p < 0.001; Student’s *t*-test. Scale bar: 100 μm.

Seeking to identify downstream target(s) of ERK1/2 signaling in airway epithelial cells, we set up a mesenchyme-free lung endoderm culture system in which FGF10 is the only supplemented growth factor. Distal lung endoderm buds were dissected out from whole lungs at E11.5 and were subsequently cultured with serum-free medium supplemented only with FGF10 (800 ng/ml) (Fig. 2A). Based on our observation of both morphology and cell behavior, we defined the developmental process of the *in vitro* cultured endoderm explants into two stages: (i) within the initial 24 h of the culture period (“pre-budding stage”), the lung endoderm bud became sealed, grew and expanded into a cyst, progressing toward bud formation; (ii) from 24 h to 48 h (“budding stage”), the lung endoderm underwent branching and formed many buds at the cyst surfaces (Fig. 2B). We noted that the phosphorylation level of ERK1/2 was significantly increased at 24 h and at 48 h as compared to 0 h in the cultured lung endoderm explants (Fig. 2C). Similar to our finding that tip cells exhibit high p-ERK1/2 levels during planar bifurcation (Fig. 1H), we found that p-ERK1/2 levels were high in the bud tips of cultured lung endoderm explants (Fig. 2D).

Annexin proteins are highly-conserved calcium-dependent phospholipid binding proteins^22,37^ and several Annexins are known to bind with F-actin to regulate cell migration^25,28,29^. A previous study has shown that several *Annexin* family members may be downstream targets of FGF10 and may be involved in endoderm budding process^38^. Consistently, we here found that the expression levels of *Anxa* gene family members (encoding Annnexins) showed a steady increase from 0 h to 48 h in cultured lung endoderm explants (Fig. s2A). This increasing expression trend was positively correlated with increased budding activity in lung endoderm cultures, suggesting that Annexins may be downstream targets of FGF10 and, further, that these proteins may be involved in FGF10-induced lung endoderm budding process. However, only *Anxa1*, *Anxa4* and *Anxa6* showed significantly increased expression from 24 h to 48 h, the period during which lung endoderm budding occurs. Whole-mount RNA *in situ* hybridization experiments revealed that *Anxa1* is highly expressed in the stalk airway epithelium and showed that *Anxa4* is highly expressed in the distal airway epithelium; *Anxa6* is expressed in both the epithelium and the mesenchyme (Figs 2E and s2B).

Given our finding that *Anxa4* is highly expressed in the distal airway epithelium which also has high p-ERK1/2 level (Figs 1H and 2E), we next set out to investigate whether the expression of *Anxa4* is regulated by ERK1/2 signaling. We cultured wild type lung endoderm explants with DMSO or PD0325901 (inhibitor of MEK, upstream of ERK1/2) at 0 h and then analyzed the expression levels of *Anxa4* at 48 h using qPCR. We found that inhibition of ERK1/2 signaling decreased the expression of *Anxa4*; this inhibition also reduced the expression levels of *Etv4* and *Spry2*, two well-known downstream targets of ERK1/2 signaling (Figs 2F and s2C). Whole-mount *in situ* analysis of *Anxa4* in lung endoderm explants confirmed that the expression of *Anxa4* is inhibited by treatment with PD0325901 (Fig. 2G). Taken together, these results establish that the expression of *Anxa4* is regulated by ERK1/2 signaling.

### Anxa4 promotes airway progenitor cell migration

**Figure 3:**
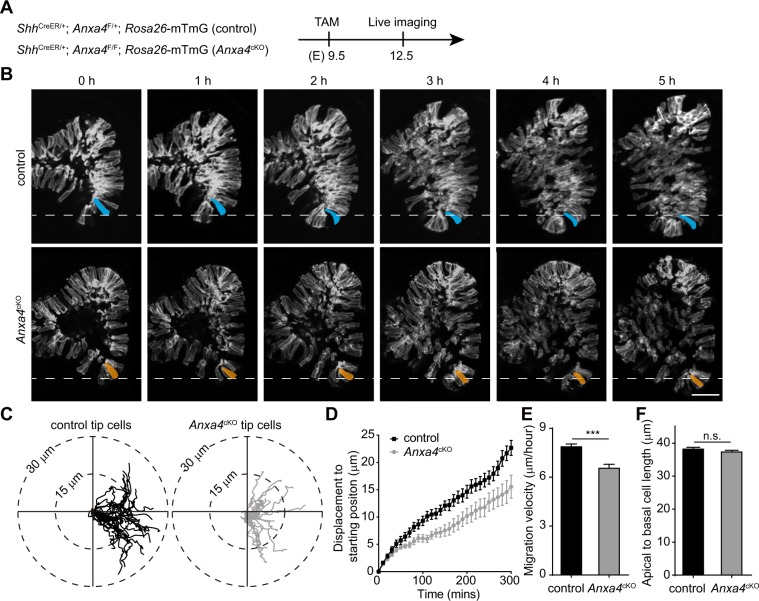
Anxa4 promotes airway epithelial progenitor cell migration. (**A**) Pregnant females carrying *Shh*^CreER/+^; *Anxa4*^F/+^; *Rosa26*-mTmG (control) and *Shh*^CreER/+^; *Anxa4*^F/F^; *Rosa26*-mTmG (*Anxa4*^*cKO*^) embryos were treated with tamoxifen (TAM) at E9.5 and lungs were imaged at E12.5. (**B**) Representative images of mosaic labeled control and *Anxa4*^*cKO*^ airway bud formation. One tip cells in control and *Anxa4*^*cKO*^ RCd.L1 buds are highlighted by blue and orange, respectively. Scale bar: 50 μm. (**C**–**F**) Cell track plots of tip cells in control lung (25 cells) and *Anxa4*^*cKO*^ lungs (16 cells) after aligning their starting positions (**C**), showing that *Anxa4*^*cKO*^ tip cells had shorter migration tracks and migration displacement than that of control tip cells (**C**,**D**); The migration velocity analysis showed that the migration velocity of *Anxa4*^*cKO*^ cells was decreased significantly (**E**) and the apical-to-basal cell length of *Anxa4*^*cKO*^ cells was similar to that of control cells (**F**). Data are presented as mean ± SEM; n = 3 samples per genotype; n.s., not significant; ***p < 0.001; Student’s *t*-test.

To gain insight into the function of Anxa4 during airway bud formation *in vivo*, we generated a *Anxa4* flox mouse line (Fig. s3A). We next investigated the effect of the loss of *Anxa4* on cell migration *in vivo* by generating pregnant female mice carrying *Shh*^*CreER/*+^; *Anxa4*^*F/*+^; *Rosa26-*mTmG (control) and *Shh*^*CreER/*+^; *Anxa4*^*F/F*^; *Rosa26-*mTmG (*Anxa4*^*cKO*^) embryos. After a tamoxifen injection at E9.5, we live-imaged the RCd.L1 bud tips of both control and *Anxa4*^*cKO*^ lungs at E12.5 (Fig. 3A). By comparing the cell migration of *Anxa4*^+*/−*^;GFP^+^ cells in *Shh*^*CreER/*+^; *Anxa4*^*F/*+^; *Rosa26-*mTmG lungs with *Anxa4*^+*/*+^;GFP^+^ cells in *Shh*^*CreER/*+^; *Rosa26-*mTmG lungs, we found that *Anxa4*^+*/−*^ cells and *Anxa4*^+*/*+^ cells shows no significant difference in cell migration displacement and migration velocity (data not shown), suggesting that loss of one copy of *Anxa4* does not impair airway epithelial cell migration. We therefore used *Shh*^CreER/+^; *Anxa4*^F/+^; *Rosa26*-mTmG lung as control in the following experiments.

After live imaging the airway progenitor cells in the RCd.L1 bud tips of both control and *Anxa4*^*cKO*^ lungs, we found that both control and *Anxa4*^*cKO*^ lung buds are able to grow and to form two new buds (Fig. 3B and Supplementary Video 3 and 4). However, the migration tracks of tip cells of control and *Anxa4*^*cKO*^ lungs revealed that the tip cells of *Anxa4*^*cKO*^ lungs have shorter migration tracks and lesser displacement as compared to tip cells of control lungs (Fig. 3C,D). Analysis of cell migration velocity showed that tip cells of *Anxa4*^*cKO*^ lungs migrate slower than did tip cells of control lungs (Fig. 3E). However, the apical-to-basal cell length of tip cells of *Anxa4*^*cKO*^ lungs is similar to that of tip cells of control lungs (Fig. 3F), suggesting that loss of *Anxa4* does not affect the tip cell shape change. These results demonstrate that *Anxa4* is required for airway epithelial progenitor cell migration during airway bud formation.

### Loss of *Anxa4* negatively affects distal airway epithelial cell fate specification

**Figure 4:**
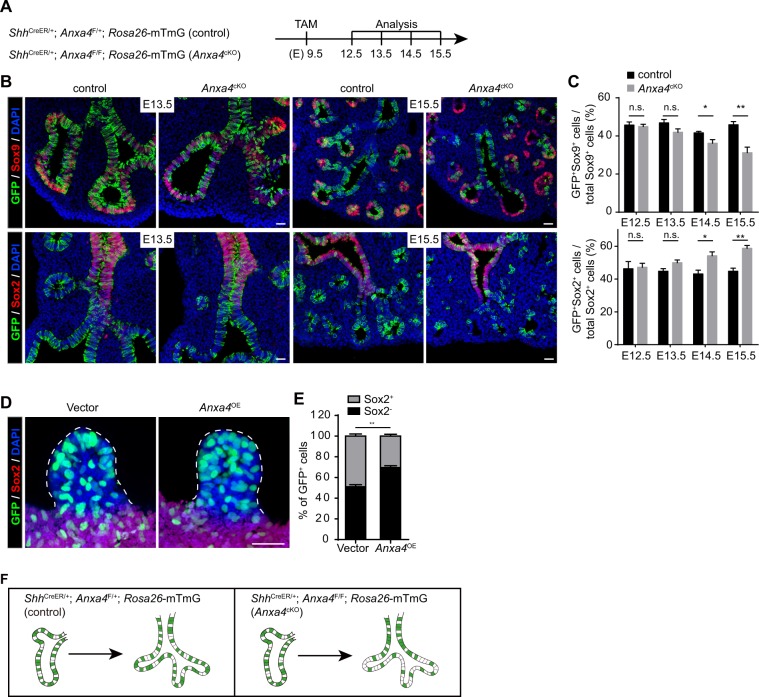
Anxa4 promotes distal airway epithelial cell fate commitment. (**A**) Pregnant females carrying *Shh*^CreER/+^; *Anxa4*^F/+^; *Rosa26*-mTmG (control) and *Shh*^CreER/+^; *Anxa4*^F/F^; *Rosa26*-mTmG (*Anxa4*^*cKO*^) embryos were treated with tamoxifen (TAM) at E9.5 and lungs were analyzed at different time point from E12.5 to E15.5. (**B**,**C**) Lungs at different time points from E12.5 to E15.5 were double stained for antibodies against GFP (green) and Sox9 (red), or GFP (green) and Sox2 (red) (**B**). Note that the proportion of GFP^+^ cells in distal airways (Sox9^+^) of *Anxa4*^*cKO*^ lungs decreased over time as compared to that of control lungs, while the proportion of GFP^+^ cells in stalk airways (Sox2^+^) of *Anxa4*^*cKO*^ lungs increased over time (**C**). Data are presented as mean ± S.E.M, n = 4. n.s., not significant; *p < 0.05; **p < 0.01; Student’s t-test. Scale bar: 25 μm. (**D**,**E**) GFP (green) and Sox2 (red) staining in cultured whole lung endoderm explants infected with vector or *Anxa4*^*OE*^ lentivirus (**D**). GFP is a reporter used to indicate lentivirus-infected epithelial cells. More GFP^+^ cells were located in the bud tips (Sox2^−^) in *Anxa4*^*OE*^ lung endoderm explants than in control explants (**E**). Data are presented as mean ± S.E.M, n = 3. **p < 0.01; Student’s *t*-test. Scale bar: 25 μm. (**F**) A model illustrating the effect of loss of *Anxa4* on epithelial cell behavior and cell fate. At E11.5, in both control and *Anxa4*^*cKO*^ lung, mosaic labeled GFP cells are evenly distributed along the airways. Four days later (E15.5), in newly formed airways of control lung, GFP^+^ cells are still evenly distributed along the airways; however, in the newly formed airways of *Anxa4*^*cKO*^ lung, GFP^+^ (*Anxa4*^−/−^) cells tend to lag behind and contribute to the stalk airways.

As *Anxa4*^−/−^ airway progenitor cells migrate slower than wild type cells, we next assessed the ability of *Anxa4*^−/−^ airway progenitor cells to become Sox9^+^ distal airway epithelial cells. Pregnant females carrying both control and *Anxa4*^*cKO*^ embryos were injected with TAM at E9.5 and these embryos were sampled at different time points from E12.5 to E15.5 (Fig. 4A). Distal and stalk airway epithelial cells were distinguished based on the expression of Sox9 and Sox2, respectively. The proportions of GFP^+^ cells in the distal airways (Sox9^+^) of *Anxa4*^*cKO*^ lungs decreased over time as compared to that of control lungs, while the proportions of GFP^+^ cells in the stalk airways (Sox2^+^) of *Anxa4*^*cKO*^ lungs increased: at E12.5, the proportions of GFP^+^ cells in the distal airways and stalk airways of *Anxa4*^*cKO*^ lungs were similar to that of control lungs; at E13.5, the proportion of GFP^+^ cells in the distal airways (Sox9^+^) of *Anxa4*^*cKO*^ lungs slightly decreased as compared to that of control lungs; at E14.5 and E15.5, the proportion of GFP^+^ cells in the distal airways of *Anxa*^cKO^ lungs significantly decreased as compared to that of control lungs (Fig. 4B,C).

We next examined the effect of loss of *Anxa4* in lung epithelial cells on cell proliferation and apoptosis using immunostaining against pH3 and Caspase3 in control and *Shh*^*Cre/*+^; *Anxa4*^*F/F*^ lungs. In *Shh*^*Cre/*+^; *Anxa4*^*F/F*^ lungs, *Anxa4* was knocked out in all airway epithelial cells. The proliferation rate of airway epithelial cells did not differ significantly between littermate control and *Shh*^*Cre/*+^; *Anxa4*^*F/F*^ lungs (Fig. s4A,B). We did not detect Caspase3^+^ cells in either control or *Shh*^*Cre/*+^; *Anxa4*^*F/F*^ lungs (Fig. s4A). The proportions of GFP^+^ airway epithelial cells to the total airway epithelial cells were similar between control and *Anxa4*^*cKO*^ lungs at both E11.5 and E15.5 (Fig. s4C–E), supporting that loss of *Anxa4* doesn’t decrease cell proliferation or induce apoptosis. Transwell migration assays with isolated primary lung epithelial cells showed that the migration of *Anxa4*^−/−^ epithelial cells toward FGF10 was significantly decreased as compared to control epithelial cells (Fig. s4F,G). These experiments demonstrated that loss of *Anxa4* decreases distal cell fate commitment and that this decrease is most likely caused by decreased cell migration, as loss of *Anxa4* does not affect cell proliferation or apoptosis.

To further investigate the role of Anxa4 in cell fate commitment, we generated an *Anxa4*-overexpression (*Anxa4*^OE^) lentivirus carrying a nuclear H2B-GFP reporter and then co-cultured lung endoderm explants with this lentivirus to overexpress *Anxa4*. After 48 h co-culture, we did whole-mount immunostaining using antibodies against GFP and Sox2. Our immunostaining results showed that more GFP^+^ cells remained in the endoderm bud tips (Sox2^−^) of *Anxa4*^OE^ endoderm explants as compared to vector-lentivirus infected endoderm explants, while fewer GFP^+^ cells were detected in the proximal endoderm (Sox2^+^) of *Anxa4*^OE^ endoderm explants (Fig. 4D,E). Taken together, our findings show that Anxa4 controls cell migration and promotes distal airway epithelial cell fate specification (Fig. 4F).

### Anxa4 functions redundantly with Anxa1 and Anxa6 in regulating endoderm budding process

**Figure 5:**
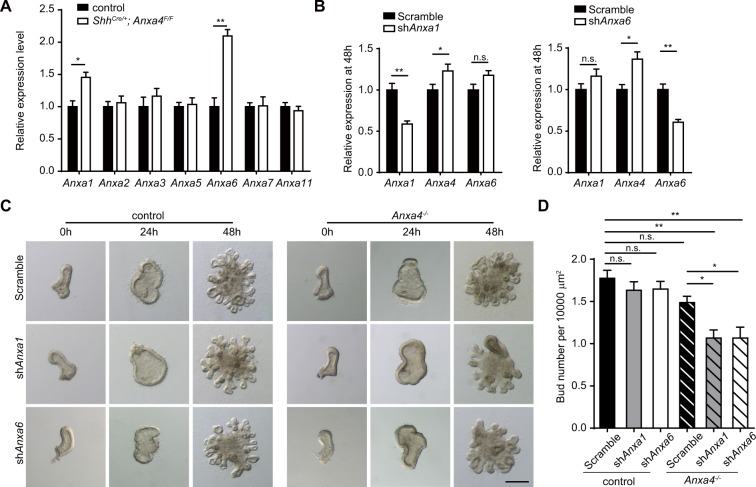
Anxa4 functions redundantly with Anxa1 and Anxa6 in regulating endoderm budding process. (**A**) Relative expression level of *Anxa1, Anxa2, Anxa3, Anxa5, Anxa6, Anxa7* and *Anxa11* in control and *Shh*^*Cre/*+^; *Anxa4*^*F/F*^ lungs. (**B**) The relative expression level of *Anxa1*, *Anxa4* and *Anxa6* at 48 h in cultured control lung endoderm treated with Scramble shRNA, or sh*Anxa1*, or sh*Anxa6*. The knockdown efficiency of sh*Anxa1* or sh*Anxa6* in cultured lung endoderm explants at 48 h was about 40%. Data are presented as mean ± S.E.M, n = 3. ***p < 0.001; Student’s *t*-test. (**C**) Representative images of cultured control or *Anxa4*^−/−^ lung endoderm explants treated with Scramble shRNA, sh*Anxa1*, or sh*Anxa6*. Scale bar: 200 μm. (**D**) Quantification of bud number of different lung endoderm groups in (B). Data are presented as mean ± S.E.M, n = 12. n.s., not significant; *p < 0.05; ***p < 0.001; Student’s *t*-test.

We next used *Shh*^Cre*/*+^ mouse line to knockout *Anxa4* in all lung epithelial cells to investigate the effect of the loss of Anxa4 on airway branching morphogenesis and found that neither the branching pattern nor the tube shape of *Shh*^*Cre/*+^; *Anxa4*^*F/F*^ mouse lungs differed significantly from their somite-matched littermate controls (Fig. s3B). We therefore hypothesized that the normal lung development phenotype that we observed in *Shh*^*Cre/*+^; *Anxa4*^*F/F*^ mice may result from functional redundancy among the Annexins^22,24^. Similar to a previous study that reported increased expression of other *Anxa* genes in *Anxa1*^−/−^ embryos^39^, we found that the expression levels of *Anxa1* and *Anxa6* were increased in *Shh*^*Cre/*+^; *Anxa4*^*F/F*^ mutant lungs (Fig. 5A). It was also notable that the expression levels of *Anxa1* and *Anxa6* increased significantly between 24 h and 48 h in lung endoderm culture (Fig. s2A).

We therefore knocked down either *Anxa1* or *Anxa6* in control and *Anxa4*^−/−^ endoderm explants with lentivirus carrying shRNA targeting *Anxa1* or *Anxa6*. The RNAi knockdown efficiency for sh*Anxa1* and for sh*Anxa6* in the lung endoderm explants was about 40% at 48 h (Fig. 5B). Note that a slight increase in the *Anxa4* expression level was detected in *Anxa1* and *Anxa6* knock-down endoderm explants (Fig. 5B). In control lung endoderm explants, knockdown of *Anxa1* or *Anxa6* by lentivirus-mediated RNAi did not impair their budding process (Fig. 5C,D). We also co-cultured *Anxa4*^−/−^ endoderm explants with Scramble shRNA lentivirus and found that *Anxa4*^−/−^ endoderm explants showed slight (not significant) decreases in the number of buds as compared with control endoderm explants (Fig. 5C,D). However, in the *Anxa4*^−/−^ endoderm explants, knockdown of *Anxa1* or *Anxa6* resulted in significantly fewer buds as compared to Scramble-shRNA-treated control or Scramble-shRNA-treated *Anxa4*^−/−^ lung endoderm explants (Fig. 5C,D). Immunostaining against pH3 and Caspase3 showed that loss of *Anxa1*, or *Anxa4*, or *Anxa6* did not impair the relative proportions of pH3^+^ and Caspase3^+^ cells in these lung endoderm explants (Fig. s5A,B), suggesting that the impaired endodermal budding process was not caused by impaired cell proliferation or by apoptosis. These experiments indicate that Anxa1, Anxa4 and Anxa6 function redundantly in regulating lung endoderm budding process.

## Discussion

Here, by combining live imaging, mouse genetics and mesenchyme-free lung endoderm culture system, we found that airway epithelial progenitor cells that migrate faster are more likely to become Sox9^+^ distal airway epithelial cells. This process is controlled by FGFR2-mediated ERK1/2 signaling. Using the lung endoderm culture system, we identified *Anxa4* as a downstream target of ERK1/2 signaling. We further demonstrated, both *in vitro* and *in vivo*, that Anxa4 promotes airway epithelial cell migration. Loss of *Anxa4* decreases airway epithelial cell migration during bud formation, while overexpression of *Anxa4* promotes cell migration. We also found that *Anxa4*^−/−^ epithelial cells tend to exhibit a lag-behind behavior and contribute to stalk airways *in vivo*, suggesting that Anxa4 plays a role in promoting distal cell fate commitment. This lag behind behavior is most likely caused by decreased cell migration, as loss of *Anxa4* does not affect cell proliferation or apoptosis.

Attempts have been made to investigate the underlying mechanisms of branching morphogenesis at cellular level over the past decades. Studies combining fluorescent reporters, mouse genetics and live imaging have revealed the dynamics and kinematics of branching morphogenesis in a variety of model organs. These studies have shown that a variety of cellular behaviors, including local proliferation, cell migration, cell invasion, apical constriction, can contribute to branching morphogenesis in different contexts^12,14,16,18^. FGF10/FGFR2 signaling is essential for cell proliferation and cell migration during lung development^6,8,36^. Studies have shown that localized cell proliferation is not required for the initiation of bud formation^12,13^. Other studies have shown that ERK1/2-signaling-controlled cell migration is involved in lung endoderm budding and renal branching morphogenesis^17,40^. In the present study, we found that cell migration is involved in airway bud formation and that tip cells migrate faster than cleft cells. Further, changes in cell shape are known to accompany cell migration^41^, we found here that tip cells are more elongated than cleft cells, a result suggesting that we are here observing active cell migration.

A previous study used microarrays to profile the transcriptomes of cultured mesenchyme-free lung endoderm explants with the goal of identifying downstream targets of FGF10/FGFR2 signaling in the lung epithelial cells during branching morphogenesis^38^. This microarray study implicated approximately 200 genes, including several members of the *Anxa* family, in the initial stages of bud formation. Consistently, we here found that the expression levels of *Anxa* family members are increased over time in lung endoderm culture and identified *Anxa4* as a downstream target of ERK1/2 signaling. We also showed that loss of *Anxa4* impairs airway epithelial cell migration, without affecting cell proliferation.

Mice deficient in *Anxa1*, *Anxa2*, *Anxa5*, or *Anxa6* have been generated and used to evaluate the physiological roles of Annexins^39,42–44^. However, all of the mice strains that lack a single Annexin are viable and exhibit normal development. It has been shown that the expression levels of other members of the *Annexin* family are altered in the tissues of *Anxa1*^−/−^ mice, suggesting the existence of reciprocal regulation between Annexin family members and of functional redundancy among Annexins^39^. Future studies that achieve the deletion of multiple *Annexin* genes will help to elucidate the precise functions of particular *Annexin* genes in embryonic lung branching morphogenesis.

## Materials and Methods

### Mice

The *Shh*^*Cre/*+ ^^45^, *Shh*^*CreER/*+ ^^45^, *Rosa26-*mTmG^46^ and *Fgfr2*^*F/F* ^^47^ mice have been described previously. All mice experiments were performed in accordance with the guidelines for the use and care of laboratory animals of the National Institute of Biological Sciences, Beijing. The experimental protocol was approved by the National Institute of Biological Sciences, Beijing (protocol number NIBS2012M0017). Mice were housed under standard environmental conditions (20–22 °C, 12–12 hr light–dark cycle) and provided food and water *ad libitum*. Animals were anesthetized by using Pentobarbital sodium before sacrifice. For live imaging experiments, pregnant females carrying *Shh*^CreER/+^; *Rosa26*-mTmG or *Shh*^CreER/+^; *Anxa4*^*F/F*^; *Rosa26*-mTmG or *Shh*^CreER/+^; *Fgfr2*^*F/F*^; *Rosa26*-mTmG were injected with one dose (30 μg/g) of Tamoxifen at E9.5. For the cell fate lineage tracing experiments, pregnant females were injected with one dose (75 μg/g) of Tamoxifen at E9.5.

### Generation and genotyping of *Anxa4*^*flox*^ mice

A conditional, replacement-type targeting vector was produced by inserting one LoxP site 210 bp upstream of *Anxa4* exon 3. A fragment containing a Loxp-flanked neo-cassette was cloned into a site 244 bp downstream of *Anxa4* exon 3. The targeting construct was linearized and electroporated into C57/BL6 ES cells and was then selected with G418 on embryonic fibroblast feeder cells. Recombinant clones containing a floxed *Anxa4* gene were identified by PCR using primers P1 (GGTGAACCATCTCTCGTCCTAAGCTCG) and P2 (CTGCTAACACATTCTCCCATCCGTCAC). The targeted clones were injected into C57BL/6J blastocysts, yielding 3 lines of chimeric mice that transmitted the *Anxa4*^*flox*^ allele through the germ line.

### Time-lapse imaging of embryonic lung explants and imaging analysis

The procedures for *ex vivo* time-lapse imaging have been described previously^48^. Briefly, lungs at E12.5 were dissected out and immediately embedded in 0.4% low melting-point agarose (Lonza) dissolved in the culture medium (1% insulin-transferrin-selenium +10 μM vitamin C and 1% penicillin/streptomycin + BGJb media). the culture dish with embedded lung explants were firstly cultured in a cell incubator (5% CO_2_, 37 °C) for 1 h, then were carried out and placed on a 37 °C heated platform for time-lapse images imaging. Time-lapse imaging was taken with a two-photon microscope (FV1000, Olympus) using a 25× water immersion objective. Imaging stacks of 512 × 512 pixels × 25 optical sections (xyzt sampling: 0.994 × 0.994 × 5 μm × 10 min) were acquired every 10 min for 5 h. For live imaging analysis, the images were opened by Imaris software, cells in the bud tip or cleft were distinguished by the end time point of live imaging: if the cells were in the two newly formed bud tips at the end time point, then we defined them as “tip cells”; if the cells were between the two newly formed bud tips at the end time point, then we defined them as “cleft cells”. We traced the xy positions of GFP^+^ tip cells and cleft cells at all time-points. All these xy position data were used for analyzed in cell migration trace plot, cell displacement to starting position and cell migration velocity.

### E-cadherin whole-mount staining

Lungs were dissected out from mouse embryos fixed with 4% PFA in PBS for 1 h at 4 °C. To facilitate the analysis of branching morphogenesis, whole lungs were stained with E-cadherin to visualize all airway epithelial cells. Whole-mount immunostaining was performed as previously described^31^.

### Lung endoderm isolation and culture

Lung endoderm isolation was performed according to Weaver *et al*.^49^. Briefly, lung explants were dissected from ICR mice at E11.5 in HBSS, washed three times in Tyrode-Ringer’s solution and then incubated in a pancreatin-trypsin solution for 5 min on ice. Lung endoderm explants were isolated from the mesenchyme using tungsten needles in DMEM/F12 media with 10% fetal bovine serum. The distal lung endoderm buds were cut off and embedded in 50% growth factor reduced Matrigel (Corning) and cultured in DMEM/F12 media supplemented with 800 ng/ml human recombinant FGF10 (Sino Biological). In some experiments, a MEK inhibitor (PD0325901, 1 μM, Selleck) was added at 1 h after culture initiation. In shRNA knockdown or *Anxa4*-overexpressing experiments, the lung endoderm was co-cultured with lentivirus added in 50% Matrigel and culture media at the time of initial culturing.

### Lentivirus production and concentration

Lentiviral shRNA plasmids corresponding to *Anxa1*, *a4* and *a6* were obtained from Sigma (Mission TRC-Mm 1.5). The corresponding TRC IDs are: *Anxa1* (TRCN0000109725, TRCN0000109728), *Anxa4* (TRCN0000110706, TRCN0000110709), *Anxa6* (TRCN0000110650, TRCN0000110653), h*Anxa4* (TRCN0000310693, TRCN0000056278). For *Anxa4*-overexpressing lentivirus, *Anxa4* was cloned into modified pCDH-CMV-MCS-PGK-H2BGFP plasmid. Lentivirus was produced by co-transfecting HEK293ft cells with a transfer vector together with the packaging vector (psPAX2) and the VSV-G envelope protein vector (pMD2.G). The supernatant containing the virus was harvested 48 h after transfection and was filtered through a 0.45 μm filter. The virus was concentrated by centrifugation at 70,000 × g for 2 h at 20 °C. The titer of the viral supernatant was determined using serial dilutions to infect NIH 3T3 cells.

### Quantitative real-time PCR

Total RNA from lung endoderm or whole lung samples was extracted using Trizol reagent (Ambion) and Direct-Zol RNA Miniprep kits (ZYMO research). RNA was reverse transcribed into first-strand complementary DNA using HiScript II Q RT SuperMix (Vazyme). The quantitative real-time PCR analysis was performed on a CFX96 Tough instrument (Bio-Rad) using KAPA SYBR FAST qPCR Master Mix (KAPA). The sequences of the primers that were used in the real-time PCR analysis are: *Anxa1*-F: AGCTTTCCTCATCTTCGCAG; *Anxa1*-R: TGGCACACTTCACGATGG; *Anxa2*-F: GTCTACTGTCCACGAAATCCTG; *Anxa2*-R: ACTCCTTTGGTCTTGACTGC; *Anxa3*-F: CTCAGATCCTCTATAATGCTGGTG; *Anxa3*-R: TGCTGTCCTCAATGTCCTTC; *Anxa4*-F: GAACTGTATGAGGCTGGAGAG; *Anxa4*-R: TGCTCTGTTCAATGTCCTTCTG; *Anxa5*-F: CGAATAGAGACCCTGATACTGC; *Anxa5*-R: ACTGCGTGTCCCAAAGATG; *Anxa6*-F: AGAGTGTCAAGAACAAGCCTC; Anxa6-R: TTCTCAATGAATTCCCTCCGG; *Anxa7*-F: TCAGATACCTCGGGACATTTTG; *Anxa7*-R: GATTCATCCGTTCCCAGTCTC; *Anxa11*-F: AATGCCTCAAGAACACCCC; *Anxa11*-R: CATCCGCTTATACTCTGCTCG; *GAPDH*-F: AAGGTCGGTGTGAACGGATTTGG; *GAPDH*-R: CGTTGAATTTGCCGTGAGTGGAG.

### Immunostaining and western blotting

Whole lung was dissected out and fixed in 4% PFA in PBS for 4 hours at 4 °C. Cultured lung endoderm explants were fixed with 4% PFA in PBS for 20 min at RT. After fixation, samples were washed twice with PBS, immersed in 30% sucrose and then embedded in OCT for cryosectioning. The primary and secondary antibodies and dilutions used were as follows: Rabbit anti-pH3 (1:250, 06–570, Millipore), Chicken anti-GFP (1:500, ab13970, Abcam), Rat anti-E-cadherin (1:100, 13–1900, Invitrogen), Rabbit anti-Caspase3 (#9664 s, CST), Rabbit anti-pERK1/2 (4370 s, CST), Goat anti-Sox2 (1:100, sc-17320, Santa Cruz); Alexa Fluor 488-Donkey anti-goat (Jackson Immuno Research), Alexa Fluor Cy3-Donkey anti-rabbit (Jackson Immuno Research), Alexa Fluor 488-Donkey anti-chicken (Jackson Immuno Research), Alexa Fluor 568-Donkey anti-rat (Jackson Immuno Research). Immunofluorescence images were taken by Lecia TCS LSI confocal. For western blotting, lungs were lysed on ice in RIPA buffer with a protease inhibitor cocktail (Roche) and PhosSTOP (Roche). The proteins were resolved via 4–20% gradient SDS-PAGE. Proteins were transferred to a PVDF membrane and incubated overnight at 4 °C and were then incubated with corresponding secondary antibody: HRP-Donkey anti-Rabbit (Jackson Immuno Research) and HRP-Donkey anti-mouse (Jackson Immuno Research), at RT for 1 h. Finally, proteins were visualized using LumiGLO chemiluminescent substrate (CST). The primary antibodies and dilutions used were as follows: rabbit anti-p-ERK/12 (1:2500, #4370, CST); rabbit anti-ERK/12 (1:1000, #9102, CST); rabbit anti-Anxa4 (1:1000, 10087–1-AP, ProteinTech); mouse anti-b-Actin (1:2500, A2228, Sigma).

### Whole-mount *in situ* hybridization

Lungs were dissected out quickly and fixed with 4% PFA in PBS for 1 h at 4 °C, washed three times with DEPC-PBS and then dehydrated through a graded methanol series in DEPC-PBS (25%, 50%, 75%, 100%, 100%) and stored at −20 °C. Whole-mount *in situ* hybridization was performed as described previously^50^. The DNA templates used for DIG-labeled probe synthesis were generated by PCR with the primers containing either the T7 or the T3 promoter. The primer sequences are: *Anxa1* 5′ primer: AATTAACCCTCACTAAAGGCCCTACCCTTCCTTCAATGTATC; *Anxa1* 3′ primer: TAATACGACTCACTATAGGCACAGAGCCACCAGGATTT; *Anxa4* 5′ primer: AATTAACCCTCACTAAAGGCAGAGGAACTTCTGCAGGTG; *Anxa4* 3′ primer: TAATACGACTCACTATAGGCAGTACTCGCTGGAACATGAA; *Anxa6* 5′ primer: AATTAACCCTCACTAAAGGCAGGAAGATGCCCAGGAAATAG; *Anxa6* 3′ primer: TAATACGACTCACTATAGGCTCAAGTCAGGCAGGGTTATG. PCR products were purified and used as templates for probe labeling. DIG-labeled RNA probes were generated using the appropriate RNA polymerase, as described in the manufacturer’s manual (Roche). The quality of labeled RNA probes was assessed by agarose gel electrophoresis. The RNA density was quantified by the ImageJ software.

### Isolation of primary lung epithelial cells and Transwell migration assays

The procedures for primary lung epithelial cell isolation have been described previously^51^. Briefly, after removing the heart and trachea, E14.5 lung lobes were minced into very small pieces and digested in DMEM media with neutral protease (Worthington) and DNase I (Roche) at 37 °C for 20 min. After stopping digestion and passing cells through 40 μm filter, we next centrifuged samples for 10 min at 350 × g, re-suspended the cell pellet in RBC lysis buffer (BD Biosciences) to remove red blood cells and centrifuged samples again for 10 min at 350 × g. The cell pellet was suspended with DMEM/F12 plus 10% FBS and cultured in 35 mm dish for 1.5 hours. The supernatant was collected and centrifuged for 3 min at 350 × g to obtain purified primary lung epithelial cells, which were suspended with DMEM/F12 plus 1% FBS and quantified (cell number) using a hemocytometer. For the Transwell® migration assays, 5 × 10^4^ cells in 200 μl were seeded into the upper well of the Transwell® apparatus (6.5 mm diameter, 8 μm pore size, Corning Costar); 600 μl of DMEM/F12 medium supplemented with 400 ng/ml human recombinant FGF10 was added into the lower chamber. Following incubation for 48 hours at 37 °C to allow cell migration, cells were fixed with 4% PFA and perform crystal violet staining.

## Electronic supplementary material

Supplementary Video 1

Supplementary Video 2

Supplementary Video 3

Supplementary Video 4

Supplementary information

## Data Availability

All data generated and analyzed during this study are either included in this publication and the Supplementary Information, or available from the corresponding author upon reasonable request.
