# Supplementary material for: Anxa4 mediated airway progenitor cell migration promotes distal epithelial cell fate specification
Source: Sci Rep. 2018 Sep 25;8:14344. doi: 10.1038/s41598-018-32494-z (PMC6156511; doi:10.1038/s41598-018-32494-z)
Supplement: Supplementary file 5 — Supplementary information [file 41598_2018_32494_MOESM5_ESM.pdf]

Anxa4 mediated airway progenitor cell migration promotes distal epithelial cell fate specification

Kewu Jiang[1,2], Zan Tang[2], Juan Li[2], Fengchao Wang[2], Nan Tang[2] *

[1]College of Life Sciences, Beijing Normal University, Beijing, 100875, China
[2]National Institute of Biological Sciences, Beijing, 102206, China
[3]College of Life Sciences, Peking University, Beijing, 100871, China

*To whom correspondence should be addressed. Email: tangnan@nibs.ac.cn

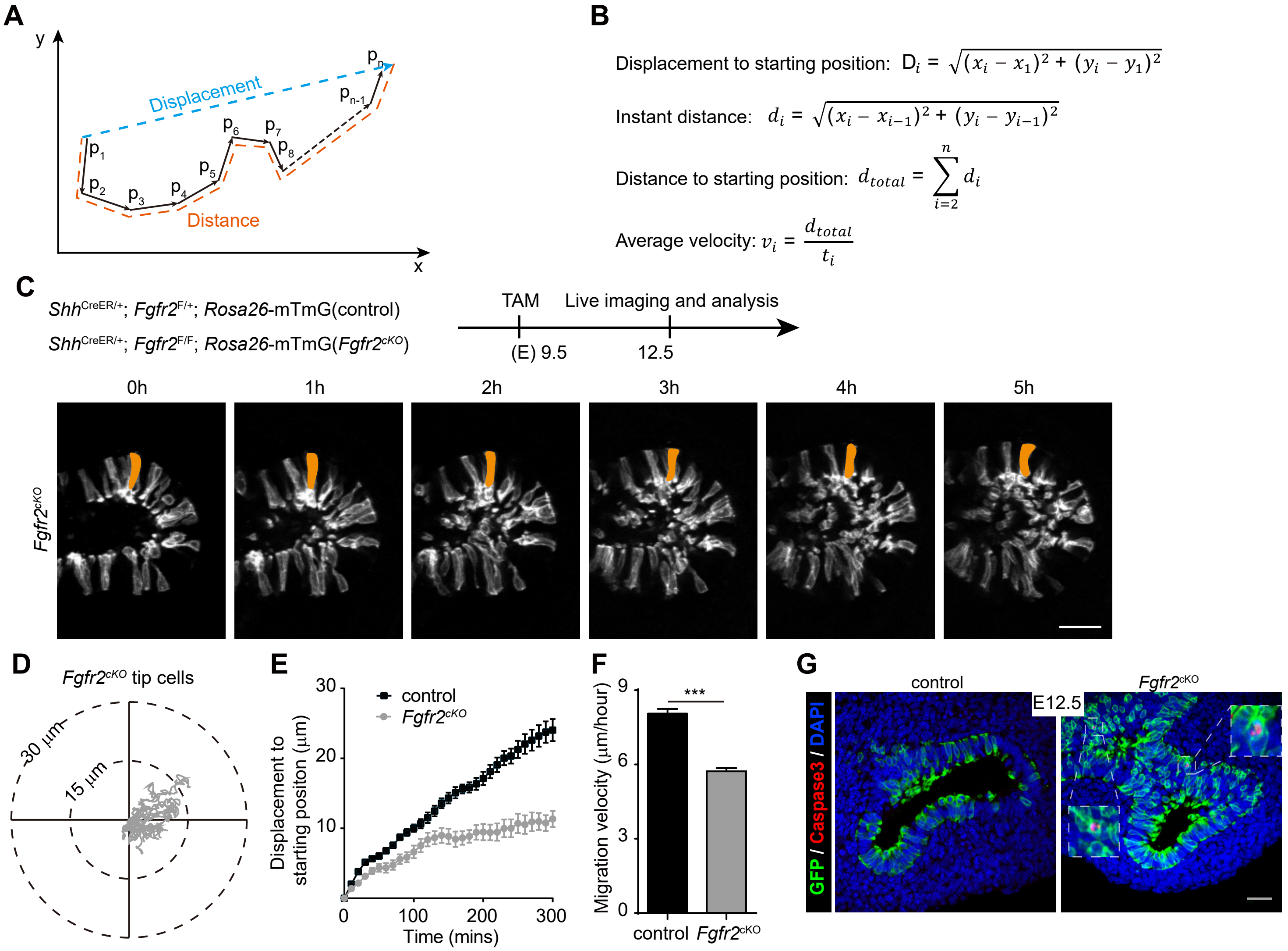

Figure s1. Loss of *Fgfr2* impairs cell migration of tip airway epithelial cells

(A) A schematic representation of recorded cell migration track consisting of N positions pi=(x_i,y_i). Final displacement is the linear distance between end position p_n and starting position p_1.

(B) The quantification formulas for individual cells.

(C) Pregnant females carrying *Shh*$^{CreER/+}$; *Fgfr2*$^{F/+}$; *Rosa26*-mTmG (control) and *Shh*$^{CreER/+}$; *Fgfr2*$^{F/F}$; *Rosa26*-mTmG (*Fgfr2*^{cKO}) embryos were treated with tamoxifen (TAM) at E9.5 and lungs were dissected out for live imaging at E12.5. Representative RCd.L1 bud tip images of *Fgfr2*^{cKO} lungs were presented. One tip cells were highlighted by Orange. Scale bar: 50 µm

(D-F) Cell track plots of tip cells (17 cells) from *Fgfr2*^{cKO} lung after aligning their starting positions (D), showing that tip cells of *Fgfr2*^{cKO} lungs had shorter migration tracks and displacement than did cleft control tip cells (D, E); The migration velocity analysis showed that tip cells of *Fgfr2*^{cKO} lungs migrate slower than control tip cells (F). Data are presented as mean ± SEM; n=3 live image samples; ***, $p<0.001$; Student's t-test.

(G) GFP (green) and Caspase3 (red) staining of lung section with the indicated genotypes at E12.5. Loss of *Fgfr2* in airway epithelial cells induced apoptosis in airway epithelial cells. Scale bar: 25 µm

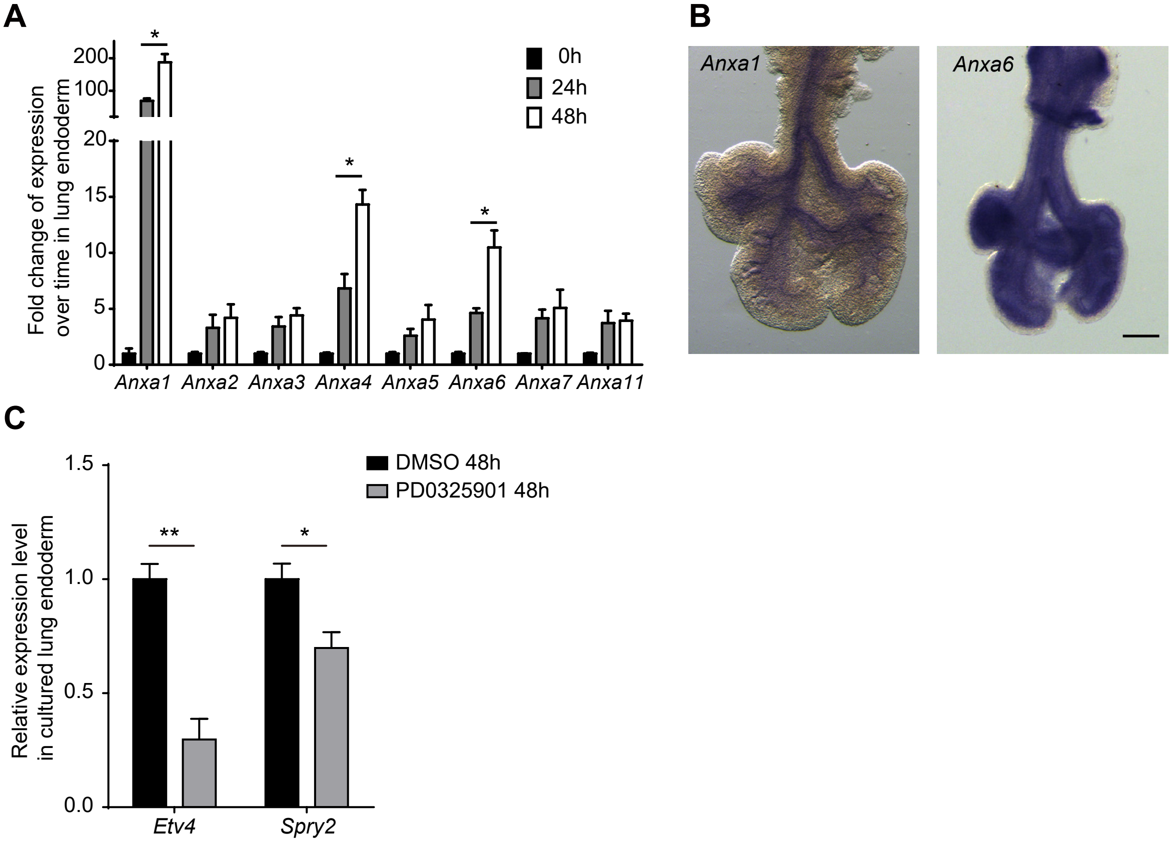

Figure s2. The expression levels of *Anxa* family members are increased in cultured lung endoderm

(A) Fold change in the expression levels of *Anxa* family members in lung endoderm quantified by qPCR (data are presented as mean±S.E.M, n=3). *, $p<0.05$; Student's t-test.

(B) Whole-mount in situ hybridization of Anxa1 and Anxa6 in WT lungs. Scale bar: 200 μm

(C) The expression levels of *Etv4* and *Spry2* at 48h in cultured lung endoderm treated with DMSO or PD0325901. The expression of *Etv4* and *Spry2* is inhibited by PD0325901. Data are presented as mean±S.E.M, n=3. *, $p<0.05$; **, $p<0.05$; Student's t-test.

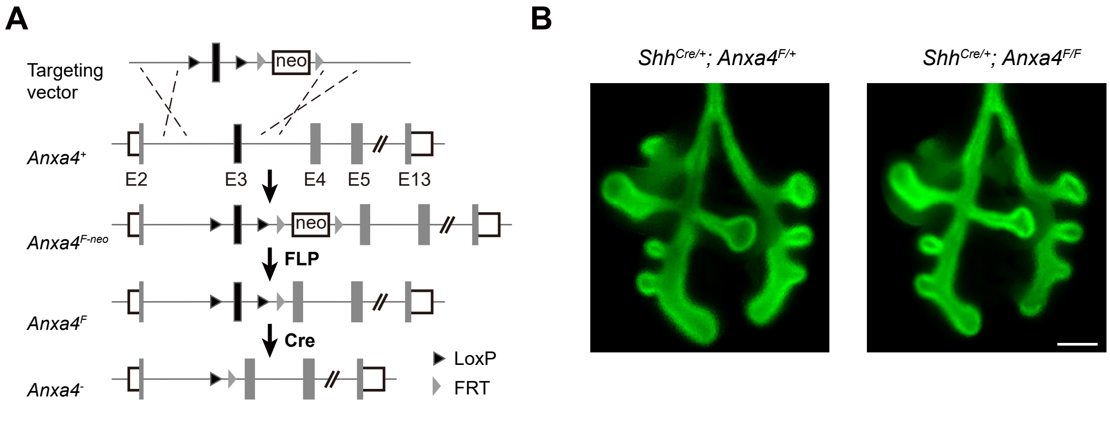

Figure s3: Loss of *Anxa4* does not affect the airway branching pattern

(A) Diagram illustrating the strategy of generating a conditional *Anxa4* and an *Anxa4* null allele. Structures of the targeting vector, *Anxa4* allele, and targeted *Anxa4* allele are shown. The *Anxa4*^{F} allele was generated by FLP-mediated DNA recombination. To generate *Anxa4*$^-$ allele, the mice carrying *Anxa4*^{F} allele were crossed with mice that express Cre.
(B) *Shh*$^{Cre/+}$;*Anxa4*$^{F/F}$ lungs showed a normal branching pattern and airway tube shape, similar to that of control lungs. Scale bar: 200 μm

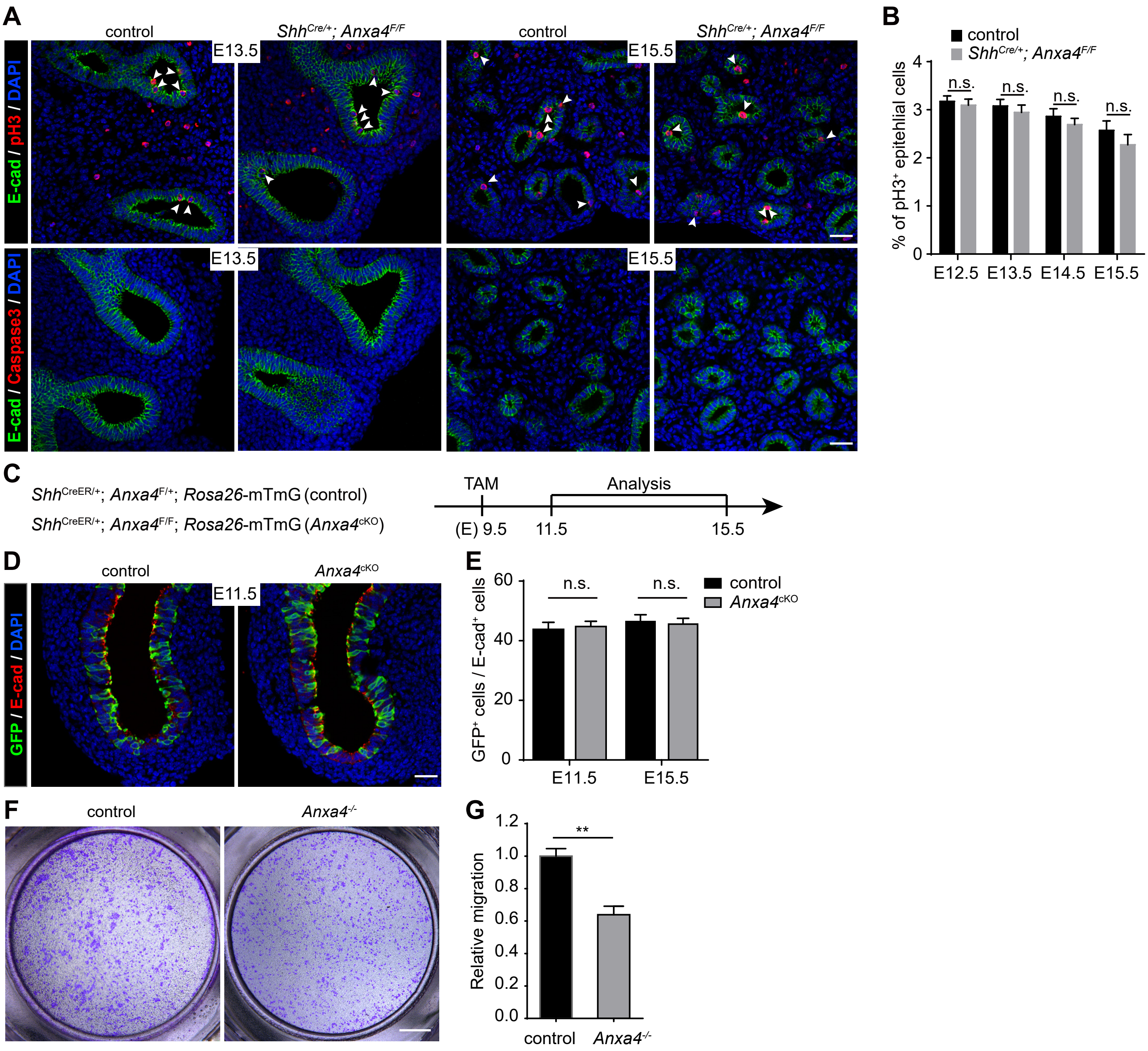

Figure s4: Loss of Anxa4 impairs airway epithelial cell migration, but does not affect cell proliferation or apoptosis

(A, B) E-cad (green) and pH3 (red), or E-cad (green) and Caspase3 (red) staining of lung section with the indicated genotypes at E13.5 and E15.5 (A). Knockout of *Anxa4* did not affect the percentage of $pH3^+$ epithelial cells (B), and no apoptosis cells were detected in either control or $Shh^{Cre/+}$;*Anxa4*$^{F/F}$ lungs (A). Data are presented as mean±S.E.M, n=3. n.s., not significant. Scale bar: 25 μm

(C) Pregnant females carrying *Shh*$^{CreER/+}$; *Anxa4*$^{F/+}$; *Rosa26*-mTmG (control) and *Shh*$^{CreER/+}$; *Anxa4*$^{F/F}$; *Rosa26*-mTmG (*Anxa4*^{cKO}) embryos were treated with tamoxifen (TAM) at E9.5 and lungs were analyzed at E11.5 and E15.5.

(D) GFP (green) and E-cad (red) staining of lung section with the indicated genotypes at E11.5, the distribution of GFP^+ cells in control and *Anxa4*^{cKO} lungs shows no obvious difference.

(E) The GFP labelling efficiency is similar in control and *Anxa4*^{cKO} lungs at both E11.5 and E15.5. Data are presented as mean±S.E.M, n=3. n.s., not significant.

(F, G) Representative images of Transwell migration assay of control and *Anxa4*$^{-/-}$ primary lung epithelial cells (F). The migration of *Anxa4*$^{-/-}$ primary lung epithelial cells was impaired significantly as compared to control primary lung epithelium (G). Data are presented as mean±S.E.M, n=3. **, p<0.01; Student's t-test. Scale bar: 1 mm

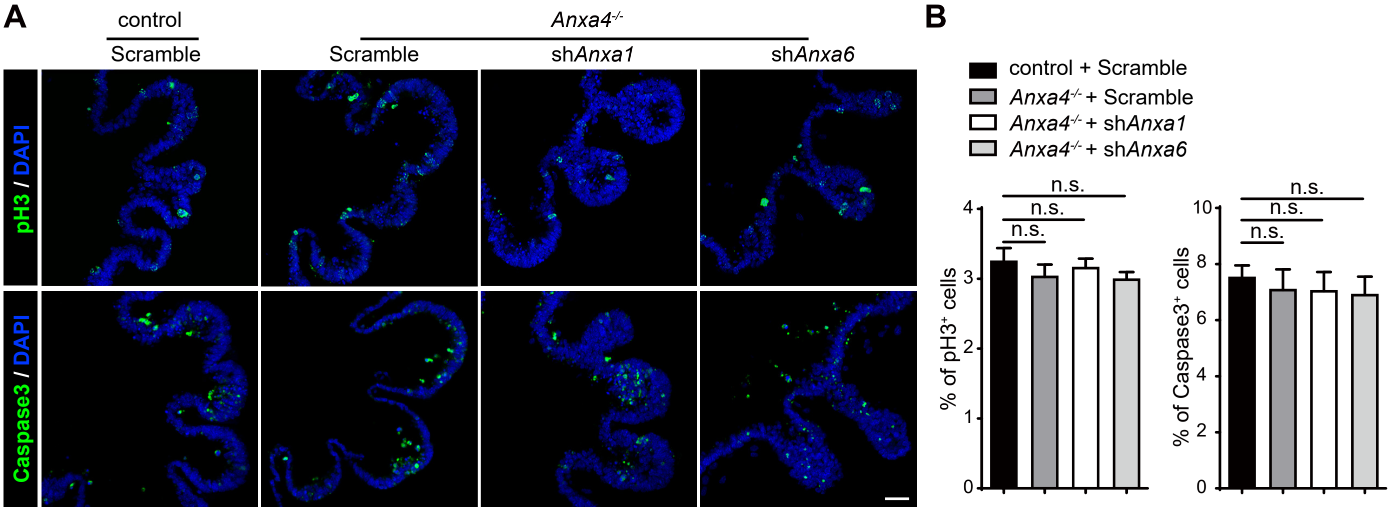

Figure s5: **The loss of *Anxa1* or *Anxa6* in *Anxa4*$^{-/-}$ endoderm does not affect cell proliferation and cell apoptosis**
(A, B) pH3 or Caspase3 staining of lung endoderm section with indicated treatment (A). Loss of *Anxa1*, or *Anxa4*, or *Anxa6* did not affect the ratio of pH3^{+} or Caspase3^{+} cells (B). Data are presented as mean±S.E.M, n=3. n.s., not significant. Scale bar: 25 μm

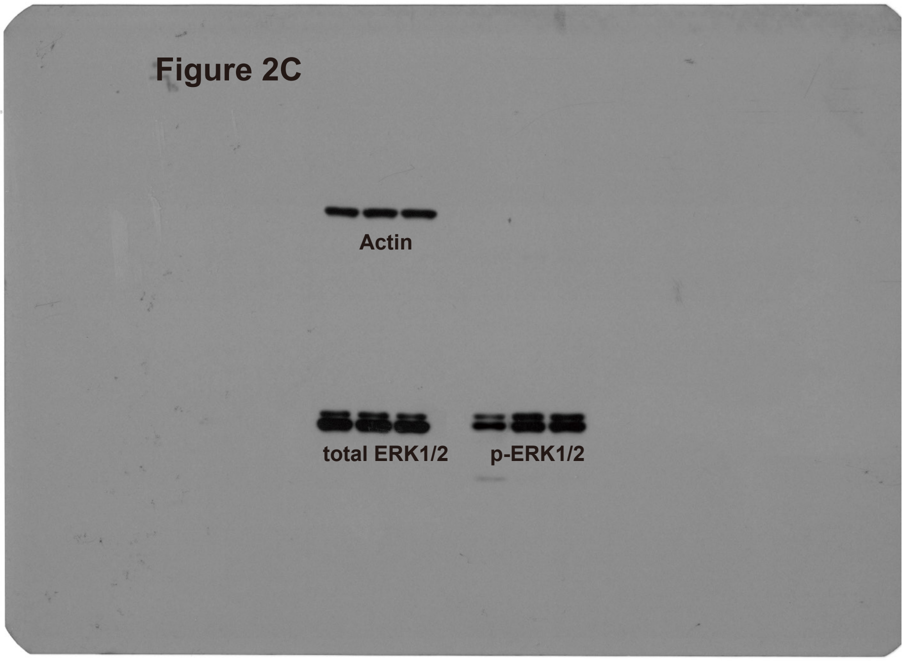

Figure s6：**Uncropped scan of western blot displayed in Fig. 2C**
